# Optimal Dimension of Peripheral Iridotomy for Anatomical Efficacy in Primary-Angle-Closure Disease

**DOI:** 10.3390/vision10020027

**Published:** 2026-05-13

**Authors:** Ludovico Alisi, Premanand Chandran, Mrunali M. Dhavalikar, Niklank Mehta, Padmavathy A. Sivakumar, Abhipsa Sahu, Rohan A. J. Daniel, Ganesh V. Raman

**Affiliations:** 1 Department of Sense Organs, Sapienza University of Rome, 00185 Rome, Italy; ludovico.alisi@uniroma1.it; 2 Glaucoma Services, Aravind Eye Hospital, Coimbatore 641014, Tamil Nadu, India; drcgprem@yahoo.co.in (P.C.); mrunali8@yahoo.co.in (M.M.D.); niklankmehta@aravind.org (N.M.); thatsme.abhipsa@gmail.com (A.S.); drrohandaniel@gmail.com (R.A.J.D.); 3 Statistics Department, Aravind Eye Hospital, Coimbatore 641014, Tamil Nadu, India; cbe.biostatistician@aravind.org

**Keywords:** laser peripheral iridotomy, angle closure disease, angle closure spectrum, glaucoma, iridotomy area

## Abstract

The aim of this study is to determine the optimal functional size of laser peripheral iridotomy (LPI) for anterior chamber parameter improvement in primary angle-closure disease (PACD). This study evaluated 109 eyes from 62 consecutive phakic patients. Baseline and one-week post-LPI anterior segment OCT were utilized to measure anterior chamber volume (ACV), anterior chamber angle (ACA), and iridotomy dimensions. Data was analyzed using linear mixed-effects models (LMMs), generalized additive models (GAMs), and receiver operating characteristic (ROC) curves. Post-LPI, significant increases occurred in ACA 500 (+7.54°), ACV (+11.09 mm^3^), and gonioscopic grade. LMMs confirmed a positive association between iridotomy size and anatomical expansion. GAMs demonstrated a saturation effect for ACV improvement, plateauing at 0.1 mm^2^ (narrow area) and 0.25–0.30 mm^2^ (superficial area), while the ACA relationship remained predominantly linear. ROC analysis identified preliminary superficial area cutoffs of 0.14 mm^2^ and 0.12 mm^2^ as discriminators of above-median volumetric and angular response, respectively. These findings suggest that LPI size is an independent determinant of anatomical response, beyond simple patency. As a preliminary, hypothesis-generating target, a superficial iridotomy area of approximately 0.12–0.14 mm^2^ was associated with above-median volumetric and angular response in this cohort. Prospective validation is required before these thresholds can be incorporated into clinical practice.

## 1. Introduction

Primary angle-closure disease (PACD) is a major global cause of irreversible vision loss. A recent meta-analysis estimates that, among adults aged 40–80 years, the number of patients affected by primary angle-closure glaucoma (PACG) will reach 32.04 million by 2040. Moreover, the prevalence of PACG shows a strong geographical polarization in Asia, underscoring a substantial regional burden [1]. Within the PACD spectrum, the International Society for Geographical and Epidemiological Ophthalmology (ISGEO) framework distinguishes primary angle-closure suspects (PACS), primary angle closure (PAC), and PACG, separating the mechanism of trabecular obstruction from the presence of glaucomatous damage; this classification emphasized that many individuals with iridotrabecular contact have no established neuropathy but remain at risk [2]. Mechanistically, angle closure is heterogeneous: pupillary block, iris and ciliary body configuration, lens position, and vault all contribute, reinforcing the need for individualized anatomic assessment [3]. Laser peripheral iridotomy (LPI) has been the standard first-line intervention for PACD for more than 50 years and remains a common procedure [4]. High-quality evidence suggests only a modest prophylactic effect in PACS. In the Zhongshan Angle-Closure Prevention (ZAP) randomized trial, incident PAC endpoints occurred at 4.19 vs. 7.97 per 1000 eye-years in treated versus untreated fellow eyes, with low absolute event rates limiting the population benefit of routine prophylaxis [5]. These data highlight both the scale of the at-risk population and the ongoing uncertainty regarding the optimal use of LPI to maximize clinically significant outcomes. A key unresolved question is: how large should the iridotomy be? A contemporary expert argues that an LPI of at least 200 µm is generally advisable, with larger openings required in specific contexts, such as uveitic glaucoma [6]. Historical modelling and clinical observations likewise suggest a minimum effective diameter in the 150–200 µm range to meaningfully reduce the anterior–posterior chamber pressure differential that drives iris bombe [7]. Despite widespread use, real-world data on size–efficacy correlations are missing. Therefore, the present study aims to bridge this knowledge gap by quantitatively correlating LPI size with anatomical outcomes to suggest, data-driven efficacy cutoffs.

## 2. Materials and Methods

The present retrospective observational, single-center cohort study was conducted from an analysis of the clinical records at Aravind Eye Hospital in Coimbatore, Tamil Nadu, India. Consecutive phakic patients undergoing LPI for any of the angle-closure disease spectrum (PACS, PAC, and PACG according to previously published criteria) [2] between July 2025 and August 2025 were recruited. The study was conducted in accordance with the Declaration of Helsinki and obtained approval from the local Institutional Review Board (RET202500518).

Inclusion criteria were age 40–80 years; Shaffer grade < 2 in ≥2 quadrants on dark-room gonioscopy with 4 mirror goniolens; clear media permitting OCT imaging; patent LPI at one week observation; and PAC and PACG patients were included only if no peripheral anterior synechiae were visible on gonioscopic assessment. Exclusion criteria were prior ocular surgery or laser, secondary angle closure, uveitis, trauma, dense cataract (LOCS III nuclear > 3), or poor fixation.

All patients underwent a baseline visit (t0) with IOP measurement and assessment of gonioscopic score. Before the iridotomy, all patients underwent anterior segment (AS)- OCT (ANTERION, Heidelberg Engineering, Heidelberg, Germany) to assess anterior chamber parameters. A specialized technician acquired AS-OCT scans (radial 6-line HD protocol, in mesopic conditions). Collected data included: anterior chamber depth (ACD), aqueous depth (AQD), anterior chamber angle at 500 µm (ACA500), angle opening distance at 500 µm (AOD500), trabecular-iris space area at 500 µm (TISA500) on the horizontal meridian (0–180°), anterior chamber volume (ACV), lens thickness (LT) and lens vault (LV).

**Figure 1:**
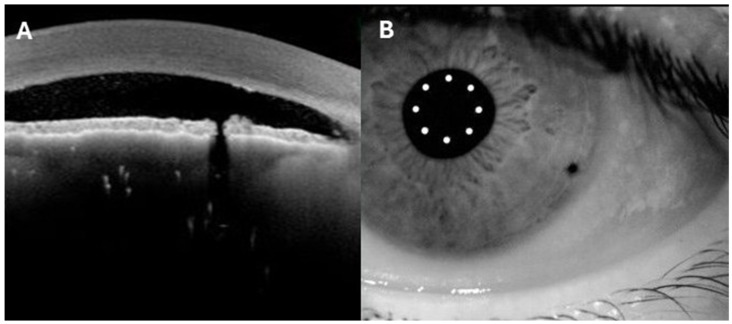
Illustrative representations of the narrow area (**A**) and the superficial area (**B**) in AS-OCT images.

On the same day, patients underwent a single Nd:YAG LPI after 3 drops of pilocarpine 15 min apart. Patency was confirmed immediately and at 1 h by retro-illumination and beam splitting. After 1 h on the same day, each patient underwent a second AS-OCT with the metrics setting to evaluate the opening (t1). Data on the central diameters of LPI were collected for further evaluation. The same protocol was repeated at 1 week (t2), including gonioscopic values, IOP, and AS-OCT. Gonioscopy values were summarized as the sum of all 4 quadrants (0–16) as previously reported [8]. AS-OCT parameters were averaged between the nasal and temporal quadrants. Superficial area of the iridotomy and area at the narrowest point were also measured at both timepoints (Figure 1A,B). Superficial area was calculated from AS-OCT images with the freehand measurement from ImageJ software Ver. 1.54 (National Institutes of Health, Bethesda, MD, USA). The narrow area was measured as follows: calipers measure two orthogonal diameters of the iridotomy, the horizontal (Dh) and vertical (Dv) axes, in micrometres. The narrow area was calculated assuming an elliptical (oval) shape: Area PI = π (Dh/2) (Dv/2) (values expressed in μm^2^). Measurements were conducted with ImageJ software Ver. 1.54 as well.

### Statistical Analysis

All statistical analyses were performed using R Studio statistical software Ver 4.5.3 (Integrated Development Environment for R. Posit Software, PBC, Boston, MA, USA). A two-sided *p*-value of < 0.05 was considered statistically significant. Descriptive statistics were calculated for patient demographics and baseline ocular parameters. Continuous variables were summarized using means and standard deviations (SDs), while categorical variables were described by frequencies and percentages. To assess the overall effect of the laser peripheral iridotomy, changes in key anterior chamber parameters (ACD, ACA, AOD, TISA, ACV, and gonioscopy grade) between baseline and the one-week follow-up were analysed using paired t-tests (Δ values, calculated as 1-week follow-up minus baseline). Based on the a priori clinical hypothesis that the effective post-procedural iridotomy size—rather than its acute, immediately post-laser appearance—drives the early anatomical response to LPI, the primary predictor variables were pre-specified as the LPI superficial area and the LPI narrow area, both measured at the 1-week timepoint (t2). The choice of t2 as the reference timepoint was made a priori on biological grounds: it reflects the stable iridotomy dimension after the resolution of acute pharmacological effects (pilocarpine-induced miosis) and post-procedural iris edema and is contemporaneous with the primary anatomical outcomes. The primary anatomical outcomes were pre-specified as the change between baseline and 1 week in anterior chamber volume (ΔACV) and anterior chamber angle at 500 µm (ΔACA), based on their documented sensitivity to LPI. Pearson correlation coefficients between iridotomy dimensions and anatomical changes were computed as descriptive bivariate effect sizes; analogous correlations using iridotomy dimensions at 1 h (t1) were performed as a sensitivity analysis to support the a priori choice of t2. To account for the non-independence of measurements from the two eyes of the same patient, linear mixed-effects models (LMMs) were subsequently employed. These models were used to determine the effect of iridotomy size (both superficial and narrow) on the ΔACV and angle ΔACA, with patient ID included as a random intercept to control for inter-subject variability. Model assumptions of normality and homoscedasticity of residuals were assessed graphically. To further explore the dose–response relationship between iridotomy size and the magnitude of anatomical change, the assumption of linearity was formally tested. We hypothesized that there would be a saturation or plateau effect.

To investigate if the effect of iridotomy size might exhibit a non-linear pattern, generalized additive models (GAMs) were fitted. For each of the four primary relationships, a GAM was constructed with the anatomical change as the dependent variable and the iridotomy size metric included as a penalized regression spline. Evidence for a significant non-linear relationship was determined based on two outputs from the model summary: the statistical significance of the smooth term and an estimated degrees of freedom (edf) value substantially greater than 1, indicating that the model favoured a curved line over a straight line. To identify a clinically relevant cutoff for iridotomy size, receiver operating characteristic (ROC) curve analysis was performed. The primary outcome for this analysis was defined as “anatomical success”, operationalized as a relative change in ACV or ACA greater than the sample median (success = ΔACA/baseline ACA500). This relative metric was specifically chosen over an absolute change cutoff to account for the wide variability in baseline angular width, ensuring that a significant proportional improvement in a very narrow angle was appropriately classified as ‘success’.

The Area under the curve (AUC) was calculated to assess the predictive power of both the iridotomy’s narrowest area and its total superficial area. The optimal cutoff value was determined as the threshold that maximized the sum of sensitivity and specificity (Youden’s J statistic). To validate the manual AS-OCT morphometric measurements, a reliability analysis was performed on a randomly selected subset of 30 eyes. Intra-observer reliability was calculated using a two-way mixed-effects model for absolute agreement (ICC 3,1), treating the rater as a fixed effect. Inter-observer reliability was assessed using a two-way random-effects model for absolute agreement (ICC 2,1), treating both raters and subjects as random effects. ICC values were interpreted according to the guidelines by Koo and Li [9]: <0.50 (poor), 0.50–0.75 (moderate), 0.75–0.90 (good), and >0.90 (excellent).

## 3. Results

The study included 109 eyes from 62 patients (56.9% female) with a mean age of 57.44 ± 10.65 years. PACS accounted for 77 eyes (70.6%), PAC for 21 eyes (19.3%), and PACG for 11 eyes (10.1%). Clinical characteristics are summarized in Table 1. One week following laser peripheral iridotomy, there were statistically significant increases in ACA500 (mean change +7.54°, *p* < 0.001), ACV (mean change +11.09 mm^3^, *p* < 0.001), AOD (mean change 0.04 mm, *p* < 0.001), TISA500 (mean change 0.02 mm^2^, *p* < 0.001), and gonioscopic grade (mean change +4.92 units, *p* < 0.001). All the other changes in the studied parameters were not statistically significant. Reliability analysis was performed to validate the manual AS-OCT measurements. The LPI narrow area demonstrated excellent intra-observer repeatability (ICC = 0.97; 95% CI: 0.93–0.98) and good inter-observer reproducibility (ICC = 0.87; 95% CI: 0.74–0.93). The LPI superficial area, obtained via freehand tracing, showed good intra-observer reliability (ICC = 0.87; 95% CI: 0.75–0.94) but moderate inter-observer agreement (ICC = 0.71; 95% CI: 0.47–0.85).

### 3.1. Exploratory Analysis

Areas showed a considerable shrinking between t1 (1 h after LPI) and t2 (1 week after LPI). Mean superficial area t1 was 0.244 ± 0.153 mm^2^ and 0.155 ± 0.137 mm^2^ at t2 (*p* < 0.001). The narrow area showed a similar behaviour, starting at 0.106 ± 0.122 mm^2^ at t1 and reaching 0.054 ± 0.079 mm^2^ at t2 (*p* < 0.001). To quantify the typical shrinkage experienced by each iridotomy, the relative reduction was calculated for each eye individually. On average, the superficial area decreased by 36.7% ± 45.4%, and the narrowest functional area decreased by 21.4% ± 86.6% during the first week. The large standard deviation indicates a high variability in the shrinkage process among different eyes.

Bivariate Pearson correlations between the pre-specified predictors (narrow and superficial areas at t2) and the pre-specified outcomes confirmed positive associations consistent with the a priori clinical hypothesis. The narrow iridotomy area at t2 correlated with ΔACV (r = 0.44, *p* < 0.001) and ΔACA (r = 0.27, *p* = 0.004); the superficial area at t2 correlated with ΔACV (r = 0.39, *p* < 0.001) and ΔACA (r = 0.22, *p* = 0.02). The pre-planned sensitivity analysis using iridotomy dimensions measured at 1 h (t1) yielded no statistically significant correlations with any anatomical outcome at 1 week, supporting the a priori biological rationale for selecting t2 (the post-shrinkage, post-inflammatory iridotomy dimension) as the reference timepoint. The marked between-eye variability in the early shrinkage process (described above) provides the mechanistic basis for this dissociation between t1 and t2 measurements.

Baseline morphological parameters such as lens vault and lens thickness were intentionally not included as covariates in these models to maintain focus on iridotomy size and to prevent statistical overfitting, which is further addressed in the study limitations.

### 3.2. Assessing the Primary Relationship Between Final Iridotomy Size and Anatomical Outcomes

To assess the primary relationship between final iridotomy size and anatomical outcomes, while controlling for the non-independence of fellow eyes, linear mixed-effects models were employed. The model revealed a highly significant and strong positive association between the narrow area and ΔACV. The coefficient for the narrow area was 33.67 (*p* < 0.001). This indicates that for every 0.1 mm^2^ increase in the narrowest area of the iridotomy, the ΔACV was estimated to increase by an additional 3.37 mm^3^. A similar significant, positive relationship was observed for ΔACA. The coefficient for the narrow area was 25.10 (*p* = 0.006). Scaled for interpretation, this result corresponds to an estimated increase of 2.5 degrees in the ACA for every 0.1 mm^2^ increase in the narrowest patent area. This confirms that iridotomy size is a robust predictor of the magnitude of angular widening. To enhance clinical applicability, the same models were run using the more easily measured total superficial area as the predictor. The superficial area was also a highly significant predictor of ΔACV. The model yielded a coefficient of 17.09 (*p* < 0.001). This corresponds to an estimated increase of 1.7 mm^3^ in ACV for every 0.1 mm^2^ increase in superficial area. The relationship remained statistically significant for ΔACA. The model yielded a coefficient of 11.48 (*p* = 0.031), corresponding to an estimated increase of 1.1 degrees in the ACA for every 0.1 mm^2^ increase in superficial area (Table 2).

### 3.3. Generalized Additive Models Test for Non-Linearity

**Figure 2:**
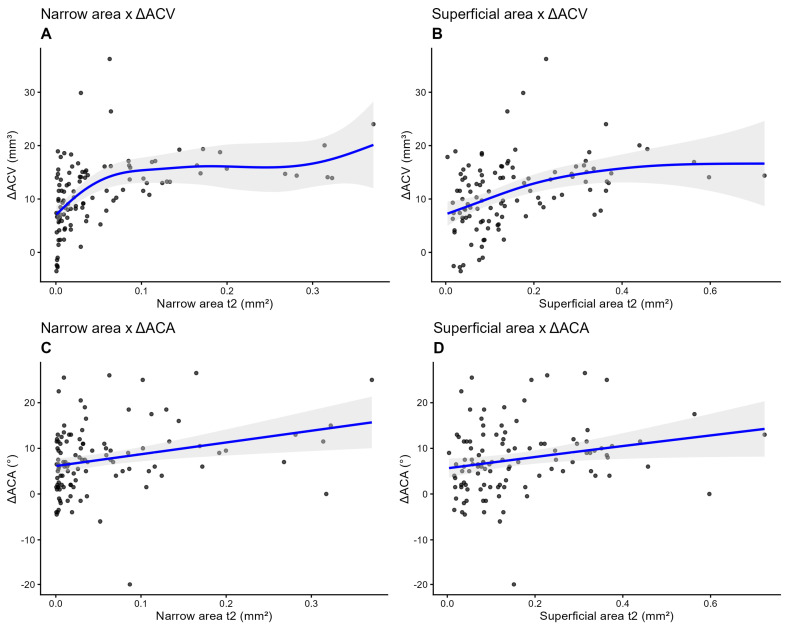
Generalized additive models for the assessment of non-linearity between narrow area and ΔACV (**A**); superficial area and ΔACV (**B**); narrow area and ΔACA (**C**); superficial area and ΔACA (**D**). ACA: anterior chamber angle; ACV: anterior chamber volume.

To further probe the nature of these relationships, GAMs were employed to test for non-linearity. The relationships between both the narrow area and superficial area and ΔACV were found to be significantly non-linear (*p* < 0.001 for both smooth terms). The estimated degrees of freedom were 3.86 for the narrow area and 1.93 for the superficial area, confirming a curvilinear association. Visual inspection of the smoothing curves revealed a clear saturation effect: a steep, positive relationship for smaller iridotomy areas, which progressively flattened into a plateau. This indicates a point of diminishing returns, beyond which a larger iridotomy yields minimal additional benefit in volumetric expansion. Visual analysis of the smoothing curve for the narrowest area suggests that the therapeutic plateau begins at approximately 0.1 mm^2^ (Figure 2A). Similarly, for the superficial area, the plateau appears to begin in the range of 0.25 to 0.30 mm^2^ (Figure 2B). Creating an iridotomy larger than these thresholds appears to yield minimal or no additional benefit regarding the volumetric expansion of the anterior chamber. In contrast, the relationships between both the narrow area and superficial area and ΔACA were found to be predominantly linear. The GAMs yielded an estimated degrees of freedom of 1.0 for the narrow area and 1.03 for the superficial area. While the associations were statistically significant (*p* = 0.004 and *p* = 0.022, respectively) (Figure 2C,D), the models confirmed that a simple linear “bigger is better” paradigm adequately describes the impact of iridotomy size on angular widening, without evidence of a saturation plateau within the range of data observed.

### 3.4. ROC Curve Analysis: Identifying a Clinical Cutoff for Anatomical Success

**Figure 3:**
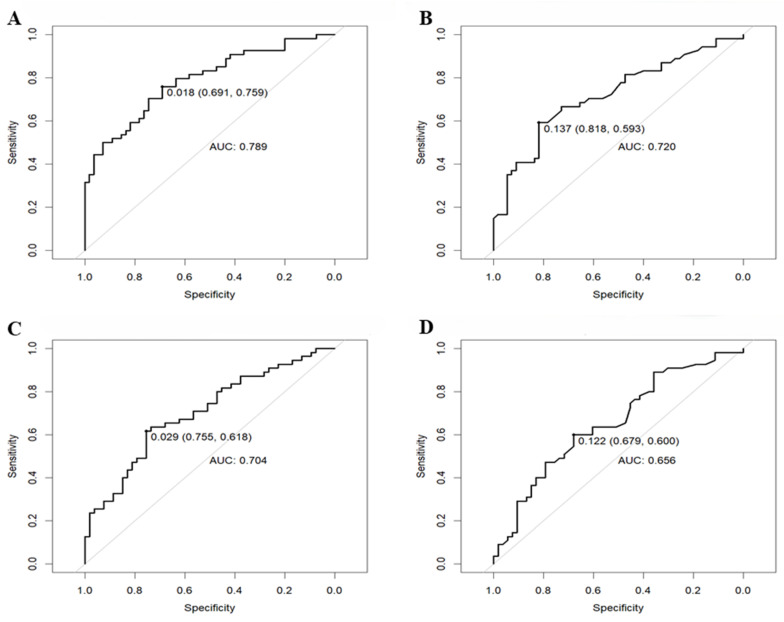
Receiver operating characteristics curves for volumetric success and narrow area (**A**) and superficial area (**B**); angular success and narrow area (**C**) and superficial area (**D**). AUC: area under the curve.

To translate these findings into clinically applicable thresholds, ROC curve analyses were performed to determine the optimal iridotomy size for predicting a greater-than-median relative increase in ACV (“volumetric success”) and ACA (“angular success”). The iridotomy’s narrow area demonstrated good predictive power for volumetric success, yielding an AUC of 0.789. The optimal cutoff was identified at 0.018 mm^2^. At this threshold, the model achieved a sensitivity of 75.9% and a specificity of 69.1% (Figure 3A). The more clinically practical superficial area also performed well, with an AUC of 0.720. The optimal cutoff was found to be 0.14 mm^2^, which corresponded to a sensitivity of 59.3% and a high specificity of 81.8% (Figure 3B). The predictive power of iridotomy size for angular success was acceptable but lower than for volumetric success. For the narrow area, the AUC was 0.704, with an optimal cutoff at 0.029 mm^2^. This threshold provided a sensitivity of 61.8% and a specificity of 75.5% (Figure 3C). For the superficial area, the AUC was 0.656, with a corresponding cutoff of 0.12 mm^2^, yielding a sensitivity of 60.0% and a specificity of 67.9% (Figure 3D). To provide a tangible number, the volumetric success corresponded to a mean increase of 16.12 mm^3^, and the angular success corresponded to an increase of 12.71°.

## 4. Discussion

This study provides a comprehensive analysis of the relationship between LPI size and the subsequent anatomical changes in the anterior chamber. LPI was effective in improving certain key anterior chamber parameters, as previously demonstrated in several studies [10]. Moreover, we observed a considerable shrinking in both superficial and narrow areas between 1 h and 1 week after LPI. This result is most likely due to the residual effect of pilocarpine, as well as the subsidence of transient post-procedural inflammation and iris oedema. Nevertheless, it is worth noting that a shrinkage of around 40% of the initial size can occur.

A key contribution of our study is the provision of quantitative, empirical evidence for the widely cited clinical guideline of a 150–200-micron LPI diameter, a standard rooted in both early clinical experience and theoretical models [6,11]. On the other hand, in conditions with increased aqueous viscosity, such as uveitis, a larger LPI may be required, as suggested by both clinical reports and mathematical modelling [12]. Considering that a circular opening of 200-micron diameter corresponds to a patent area of approximately 0.031 mm^2^, this value closely aligns with the cutoffs for the narrowest iridotomy area (identified in our ROC analyses, 0.02–0.03 mm^2^). This convergence suggests that our data-driven findings offer appropriate validation for the established clinical rule. Our analysis helps to clarify that this historical guideline likely refers to the minimal functional diameter (in µm), which corresponds to our narrowest area cutoffs (0.02–0.03 mm^2^). In contrast, the total superficial opening requires a substantially larger optimal area of 0.12–0.14 mm^2^, which would translate to an estimated superficial circular diameter of approximately 400–420 µm.

Our initial analysis using LMMs established a significant positive association between iridotomy size at one week and the magnitude of anatomical improvement for both ACV and ACA. Our linear models quantified this, demonstrating that increases in both narrow and superficial areas translated into predictable, proportional expansions of ACV and ACA.

In our findings, while a larger LPI is generally associated with a greater therapeutic effect, the nature of this relationship is not always linear. Specifically, the impact of LPI size on the increase in ACV is non-linear, exhibiting a clear saturation plateau, whereas its effect on angular widening is predominantly linear. This finding is strongly supported by theoretical biomechanical studies. Mathematical models of aqueous humour flow, such as those by Dvoriashyna et al. and Cai et al., have demonstrated that the pressure differential across the iris is highly sensitive to the orifice size, but not in a linear fashion. These models predict that as the LPI diameter increases, the resistance to flow decreases at first, rapidly equalizing the pressure between the chambers. However, once the iridotomy is large enough to accommodate the physiological rate of aqueous production with minimal resistance, further size increases have a progressively smaller effect on the trans-iris pressure gradient. This creates a point of diminishing returns, mirroring the saturation plateau observed in our clinical data [7,13].

Our models empirically confirm this theoretical principle, showing a steep benefit for smaller areas that flattens once the iridotomy is “sufficiently patent,” a point our data suggests occurs around a narrow area of around 0.1 mm^2^ or a superficial area of around 0.25–0.30 mm^2^. In contrast, the effect on angular widening remained largely linear. This suggests that different biomechanical mechanisms may govern these two outcomes. Volumetric expansion appears to be a direct consequence of pressure equalization, a process subject to a fluid-dynamic limit as described above. Angular widening, however, may be a more secondary, mechanical reconfiguration of the iris that continues to respond to larger openings, perhaps by altering the overall structural tension within the iris tissue. A cautionary suggestion may be drawn from these different behaviours, as the linear models show that more narrow angles may need larger iridotomies.

From a clinical standpoint, this exploratory work provides preliminary data that could guide the future development of practical targets. While the narrow area proved to be the statistically superior predictor for volumetric success, the strong performance of the superficial area may suggest its use as a primary clinical marker due to its greater ease of measurement. The optimal cutoff for the superficial area of 0.14 mm^2^ for predicting volumetric success is an interesting preliminary finding that requires prospective validation. Its high specificity (81.8%) positions it as a strong negative predictive tool: an iridotomy smaller than this threshold is unlikely to yield an optimal volumetric outcome. This moves the clinical assessment of an LPI beyond a subjective judgment of ‘patency’ to an objective, size-based evaluation of ‘adequacy’, a need highlighted in literature where small but patent LPIs have failed to resolve angle closure [14].

Our methodology and results align with recent efforts to quantify LPI characteristics. In a recent work by Koçer, both ACA and ACV were significantly improved by LPI, and the author concludes that LPIs that are small relative to the pupil or positioned too peripherally are associated with diminished efficacy, emphasizing the need to calibrate both LPI size and placement in relation to pupil dimensions to optimize therapeutic outcomes [15].

This study has several limitations. Its retrospective design is one; the actual efficacy of the suggested cut-offs needs prospective data to be confirmed. Secondly, we intentionally excluded eyes with PAS, resulting in a cohort predominantly composed of PACS and PAC cases, with a low prevalence of PACG. While this exclusion was necessary to isolate the anatomical response of appositional closure from permanent structural adhesions, it limits the generalizability of our findings. In more advanced or chronic stages of PACD, where PAS permanently seal the angle, the anatomical response to LPI will inherently be blunted regardless of the iridotomy size. Therefore, our proposed “optimal dimensions” should be interpreted as targets for maximizing the relief of appositional closure and pupillary block, rather than for the management of advanced synechial disease. Moreover, measurements were taken at a single post-operative timepoint, and while this timeframe successfully captures the early tissue shrinkage and initial anatomical expansion, it fails to account for long-term dynamics. The lack of extended follow-up is a significant limitation, as the clinical stability of the angle ultimately depends on the LPI remaining patent and avoiding late-onset closure. Future longitudinal studies are required to confirm whether our proposed optimal dimensions guarantee long-term patency. Furthermore, our models did not include the mechanisms of angle closure. It is known that pupillary block, plateau iris configuration, thick peripheral iris roll, and exaggerated lens vault do not respond similarly to LPI [16,17]. Additionally, other potentially influential covariates, such as the specific anatomical location of the iridotomy, were not evaluated in our current models. Further studies with larger populations are needed to evaluate these variables. Lastly, the visual symptoms of the LPI were not collected, therefore missing the potential visual impact of larger iridotomies.

## 5. Conclusions

In conclusion, this study demonstrates that LPI size is a quantifiable and complex determinant of post-operative success. We have provided empirical evidence for a non-linear saturation effect on ACV, a finding supported by theoretical biomechanical models. We have also further supported the long-standing 200-micron guideline for the effective functional opening and, most importantly, we suggest a preliminary target for the superficial LPI area of approximately 0.12–0.14 mm^2^, which corresponds to an approximate circular diameter of 400–420 microns. These findings should encourage a shift in the clinical paradigm from simply achieving patency to obtaining an optimally sized iridotomy to maximize anatomical outcomes. Whether unmeasured variability in iridotomy dimensions may have contributed to the heterogeneity of outcomes reported in prior prophylactic LPI trials, including the low absolute event rates observed in the ZAP trial, remains an open hypothesis. Future randomized studies in PACD should consider standardized reporting and stratification by quantitative LPI dimensions, rather than relying on patency alone, to address this question.

## Figures and Tables

**Table 1:** Clinical characteristics of the recruited eyes at both timepoints and the difference between timepoints.

	Pre-Iridotomy	Post-Iridotomy	Difference	*p*-Value
IOP	17.58 ± 4.94	16.86 ± 4.11	−0.72 ± 4.32	0.083
ACD	2.78 ± 0.24	2.79 ± 0.24	0.015 ± 0.11	0.128
AQD	2.28 ± 0.26	2.29 ± 0.24	0.007 ± 0.14	0.598
ACA500	18.91 ± 8.52	26.45 ± 9.65	7.54 ± 7.46	<0.001
AOD500	0.14 ± 0.11	0.19 ± 0.08	0.04 ± 0.10	<0.001
TISA500	0.04 ± 0.03	0.06 ± 0.06	0.02 ± 0.06	<0.001
ACV	102.24 ± 19.6	113.33 ± 19.22	11.09 ± 6.54	<0.001
Gonioscopy	3.67 ± 2.58	8.59 ± 3.34	4.92 ± 3.28	<0.001
LV	0.87 ± 0.96	0.77 ± 0.21	−0.09 ± 0.91	0.270
LT	4.76 ± 0.54	4.80 ± 0.34	0.03 ± 0.42	0.410

IOP: intraocular pressure; ACD: anterior chamber depth; AQD: aqueous depth; ACA500: anterior chamber angle at 500 µm; AOD500: angle opening distance at 500 µm; TISA500: trabecular-iris space area at 500 µm; ACV: anterior chamber volume; LV: lens vault; LT: lens thickness.

**Table 2:** Summary of the linear mixed models.

Model	Coefficient	95% CI	*p*-Value
Narrow area~ΔACA	25.10	7.34–42.87	<0.001
Narrow area~ΔACV	33.67	18.73–48.60	<0.001
Superficial Area~ΔACA	11.48	1.21–21.75	0.031
Superficial Area~ΔACV	17.09	8.38–25.80	<0.001

ACA: anterior chamber angle; ACV: anterior chamber volume; CI: confidence intervals.

## Data Availability

Original data are available from the corresponding author upon reasonable request.

## Abbreviations

The following abbreviations are used in this manuscript:

ACA500	Anterior chamber angle at 500 µm
ACD	anterior chamber depth
ACV	anterior chamber volume
AOD500	angle opening distance at 500 µm
AQD	aqueous depth
edf	estimated degrees of freedom
GAMs	generalized additive models
ISGEO	International Society for Geographical and Epidemiological Ophthalmology
LPI	laser peripheral iridotomy
LMMs	linear mixed-effects models
LT	lens thickness
LV	lens vault
PAC	primary angle closure
PACD	primary angle-closure disease
PACG	primary angle-closure glaucoma
PACS	primary angle closure suspects
ROC	receiver operating characteristic
SD	standard deviation
TISA500	trabecular-iris space area at 500 µm
ZAP	Zhongshan Angle-Closure Prevention
